# Overexpression of *ERF1-V* from *Haynaldia villosa* Can Enhance the Resistance of Wheat to Powdery Mildew and Increase the Tolerance to Salt and Drought Stresses

**DOI:** 10.3389/fpls.2017.01948

**Published:** 2017-11-29

**Authors:** Liping Xing, Zhaocan Di, Wenwu Yang, Jiaqian Liu, Meina Li, Xiaojuan Wang, Chaofan Cui, Xiaoyun Wang, Xiue Wang, Ruiqi Zhang, Jin Xiao, Aizhong Cao

**Affiliations:** National Key Laboratory of Crop Genetics and Germplasm Enhancement, Cytogenetics Institute, Jiangsu Collaborative Innovation Center for Modern Crop Production, Nanjing Agricultural University, Nanjing, China

**Keywords:** transcription factor, AP2/ERF, powdery mildew, salt tolerance, drought stress, transgenic wheat

## Abstract

The APETALA 2/Ethylene-responsive element binding factor (AP2/ERF) transcription factor gene family is widely involved in the biotic and abiotic stress regulation. *Haynaldia villosa* (VV, 2*n* = 14), a wild species of wheat, is a potential gene pool for wheat improvement. *H. villosa* confers high resistance to several wheat diseases and high tolerance to some abiotic stress. In this study, *ERF1-V*, an ethylene-responsive element-binding factor gene of the AP2/ERF transcription factor gene family from wild *H. villosa*, was cloned and characterized. Sequence and phylogenetic analysis showed that *ERF1-V* is a deduced B2 type *ERF* gene. *ERF1-V* was first identified as a *Blumeria graminis* f. sp. *tritici* (*Bgt)* up-regulated gene, and later found to be induced by drought, salt and cold stresses. In responses to hormones, *ERF1-V* was up-regulated by ethylene and abscisic acid, but down-regulated by salicylic acid and jasmonic acid. Over expression of *ERF1-V* in wheat could improve resistance to powdery mildew, salt and drought stress. Chlorophyll content, malondialdehyde content, superoxide dismutase and peroxidase activity were significantly differences between the recipient Yangmai158 and the transgenic plants following salt treatment. Furthermore, the expression levels of some stress responsive genes were differences after drought or salt treatments. Although *ERF1-V* was activated by the constitutive promoter, the agronomic traits, including flowering time, plant height, effective tiller number, spikelet number per spike and grain size, did not changed significantly. *ERF1-V* is a valuable gene for wheat improvement by genetic engineering.

## Introduction

Transcription factors (TFs) have been shown to control the activation of multiple stress response genes (Meshi and Iwabuchi, 1995). APETALA 2/Ethylene-responsive element binding factor (AP2/ERF), the largest family of TFs in plants, is characterized by a highly conserved AP2/ethylene-responsive element-binding factor DNA-binding domain (BD) (Riechmann and Meyerowitz, 1998). This TF family has been identified in plants such as *APETALA2* of *Arabidopsis* (Jofuku et al., 1994) and *EREBP1* of tobacco (Ohme-Takagi and Shinshi, 1995). The AP2/ERF genes regulate downstream genes by interacting with their GCC box and/or dehydration-responsive element (DRE)/C-repeat motifs at the promoter region (Riechmann and Meyerowitz, 1998). AP2/ERF TFs play crucial roles in plant growth, development and stress response, and have been targets for molecular plant breeding (Wang et al., 2016).

With the development of sequencing technology and bioinformatics tools, a genome wide survey of AP2/ERF genes has been successfully carried out in more than 10 species, and more than 200 AP2/ERF genes have been identified in *Populus trichocarpa* (Zhuang et al., 2008), maize (Zhou et al., 2012), Chinese cabbage (Song et al., 2013) and *Brassica oleracea* (Thamilarasan et al., 2014). The AP2/ERF genes are divided into five subfamilies, including APETALA2 factor (AP2), Dehydration responsive element binding factor (DREB), Ethylene responsive element binding factor (ERF), Related to ABI3/VP (RAV) and Soloist subfamilies, based on the number and similarity of their AP2/ERFBP domains (Li et al., 2013).

Some AP2 subfamily members regulate the development of flowers and fruits (Jofuku et al., 1994; Krizek, 2009; Chung et al., 2010). A large number of RAV, DREB, and ERF subfamilies of AP2/ERF genes are involved in response to adverse environmental factors, including drought, salt, and submergence (Wang et al., 2016). However, AP2/ERF genes do not always positively regulate abiotic stress tolerance, and some AP2/ERF genes have negative regulating effects. Rice gene *OsAP23* negatively regulated salt stress tolerance in *Arabidopsis* (Zhuang et al., 2013), and *OsERF922* negatively regulated resistance to salt tolerance in rice (Liu et al., 2012).

The AP2/ERF genes may play a role in response to biotic stress caused by bacteria, virus, fungi, and herbivore attack. Tobacco gene *Tsi1* from enhanced host resistance to viral, bacterial and oomycete infection in hot pepper (Shin et al., 2002), *OsERF3* from rice played roles in herbivore-induced signaling, defense and resistance (Lu et al., 2011), *AaORA* from *Artemisia annua* positively regulated the biosynthesis of artemisinin and increased resistance to *Botrytis cinerea* in *Arabidopsis* (Lu et al., 2013), and *GmERF5* enhanced resistance to *Phytophthora sojae* in soybean (Dong et al., 2015).

Besides response to adverse environmental factors, AP2/ERF genes widely participate in different physiological processes and developmental stages. For example, *NtERF32* from tobacco is required in jasmonate-inducible nicotine biosynthesis in tobacco (Sears et al., 2014). *PtaERF003* from *Populus* has a positive effect on both adventitious and lateral root proliferation (Trupiano et al., 2013). *RAP2.11* from *Arabidopsis* can respond to low-potassium and enhanced of nutrition uptake (Kim et al., 2012). In *Medicago truncatula, EFD* is an ERF transcription factor involved in the control of nodule number and differentiation (Vernié et al., 2008), and *MtSERF1* is required for somatic embryogenesis (Mantiri et al., 2008).

AP2/ERF TFs often act as ‘regulatory genes’ to modify multiple genes through binding with different *cis*-elements of the downstream genes. These TFs can improve tolerance to several stresses simultaneously (Yang et al., 2011). Crops are often subjected to multiple abiotic and biotic stresses in field, so AP2/ERF have attracted more and more attentions and have become the hot targets in the crop biotechnology (Wang et al., 2016). Wheat, one of the most important crops in the world, is affected by powdery mildew, water deficit and soil salinity, causing huge production losses in wheat production (Conner et al., 2003; Munns et al., 2012). The complexity of stress response has limited the development of wheat by classical breeding (Ashraf, 2010). *Haynaldia villosa* (2*n* = 14, VV), a wild species of wheat, has been found to confer high resistance to some diseases, such as powdery mildew, stem rust, yellow mosaic virus, cereal cyst nematode and take-all disease. Furthermore, *H. villosa* was found to confer high tolerance to some abiotic stress, such as salt and drought stress (Blanco et al., 1983; Chen et al., 1995; Zhang et al., 2005; Qi et al., 2011). Few genes from *H. villosa* have been shown to participate in stress tolerances, especially genes which could regulate both biotic and abiotic stress resistances. The aim of this study was to identify whether the *ERF1-V* gene of the AP2/ERF family is involved in powdery mildew resistance and salt and drought stress tolerance of wheat.

## Materials and Methods

### Response of *ERF1-V* to Biotic and Abiotic Stresses, and Hormones

The Contig3867, a predicted *ERF1-V* gene, was identified as a *Bgt* induced gene in *H.* by GeneChip microarray as described by Cao et al. (2011). The leaves of *H. villosa* at the two-leaf stage were inoculated with *Bgt*, or treated with 200 μmol/L ethephon (ET), 25 μmol/L jasmonic acid (JA), 5 mmol/L salicylic acid (SA), 50 μmol/L abscisic acid (ABA), 16% (w/v) PEG–6000, 200 mmol/L NaCl or 200 mmol/L CaCl_2_ for analyzing the response of *ERF1-V* to different stresses using the real-time quantitative reverse transcription–PCR (qRT–PCR). The treated leaves were sampled at time points 0, 12, 24, 48, and 60 h after *Bgt*, ET, JA, and SA treatment and at time points as 0, 1, 6, 12, and 24 h after ABA, PEG-6000, CaCl_2_, NaCl, and 4°C treatment. Three were three independent biological replicates. Total RNA (2 μg) of each sample was synthesized to first-strand cDNA using AMV reverse transcriptase. The primers of *ERF1-V* used for qRT–PCR were *ERF1-V*-QRT-F and *ERF1-V*-QRT-R, and the primers of the control gene *Tubulin* were *Tubulin-F* and *Tubulin-R* (Supplementary Table S1). The reagents were purchased from TAKARA Co. (Japan), and the reaction was performed in an ABI 7500fast (ABI, United States).

### Gene Isolation by cDNA Library Screening

The cDNA library of *H. villosa* inoculated with *Bgt* was previously constructed, and the pooled clones were stored in the 384 well plates (Chen et al., 2007). The full length *ERF1-V* gene was isolated from the cDNA library by PCR analysis using the primers designed according to the probe Contig3867 (forward primer *ERF1-V*-F and reverse primer *ERF1-V*-R, Supplementary Table S1). The mixed plasmid DNA of cDNA library pool were isolated and used as templates, and the positive pool containing *ERF1-V* was identified using PCR screening. The mixed plasmids of the positive pool were extracted and transformed into *E. coli*, and the positive single clone containing *ERF1-V* was screened using colony PCR with the same primers.

### Structure and Phylogenetic Analysis of *ERF1-V*

The conserved domain of *ERF1-V* was predicted by Prosite^1^, and the protein structure of *ERF1-V* was predicted by SWISS-MODEL^2^. Forty ERF proteins from ten species, including *Saccharum officinarum, Solanum lycopersicum, Triticum aestivum, Glycine max, Gossypium hirsutum, Capsicum annum, Nicotiana tabacum, Thinopyrum intermedium, Arabidopsis thaliana, Orazy sativa*, were initially aligned and then used to construct the phylogenetic tree. Multiple sequence alignments were performed using ClustalX (ver.1.83), and a phylogenetic tree was constructed by the Maximum likelihood (ML) method in MEGA6 ^3^. The bootstrap test of phylogeny was performed with 1,000 replications.

### Transcriptional Activation Activity Assay of *ERF1-V*

The *ERF1-V* gene was amplified using the forward primer *ERF1-V-EcoR*I-F and the reverse primer *ERF1-V-Sma*I-R (Supplementary Table S1). The PCR product and plasmid pGBKT7 (CLONTECH Co., United States) were treated with *EcoR*I and *Sma*I enzymes (TAKARA Co., Japan), respectively, followed by ligation to construct the recombinant vector pGBKT7*:ERF1-V*. The recombinant vector and the negative control *pGBKT7* were transformed into the wild yeast cells *AH109* (CLONTECH Co., United States), respectively, and cultured in *Trp* lacking media. The clones grown in the *Trp* lacking media were then transferred to the *Trp* and *His* lacking media with X-gal to test the transcriptional activation activity.

### Wheat Transformation and Positive Transgenic Plant Identification

The *ERF1-V* gene was amplified with the forward *Sma*I-containing primer and the reverse *Sac*I-containing primer (*ERF1-V*-*Sma*I-F and *ERF1-V*-Sacl-R, Supplementary Table S1). The PCR product and the plasmid pAHC 25 (Christensen and Quail, 1996) were digested with *Sma*I and *Sac*I enzymes, followed by ligation to construct the recombinant vector pAHC:*Hv-ERF*. The transgenic plants over-expression *ERF1-V* were produced by particle bombardment of callus cultured from immature embryos of *Bgt*-susceptible variety Yangmai158 (Xing et al., 2008). The positive transgenic plants were identified by PCR analysis of the selective gene *Bar* (*Bar*-F and *Bar*-R, Supplementary Table S1) and the target gene *ERF1-V* (forward primer *Ubi*-F was located in the *Ubi*-promoter and reverse primer *ERF1-V*-TR which was located in the *ERF1-V* gene, Supplementary Table S1) in plants regenerated from the bialaphos-containing culture media. Only when both the *Bar* gene and *ERF1-V* could be amplified, the plants were considered as positive transgenic plants. The gene expression level of *ERF1-V* was further analyzed by semi-quantitative RT-PCR (*ERF1-V*-RT-F and *ERF1-V*-RT-R, Supplementary Table S1), and GMY27, GMY60, GMY88 and GMY90 were identified as *ERF1-V* over-expressed lines when the expression of *ERF1-V* in these liens were compared with that in Yanamai158. The T_1_ to T_6_ generations of these four lines were used for powdery mildew resistance evaluation, the T_5_ generation plants were used for drought and salt tolerance evaluation, and the T_5_ to T_6_ generation plants were used for agricultural traits comparison.

### Evaluation of the Powdery Mildew Resistance

The positive transgenic plants of T_1_ lines generated from the identified T_0_ individuals over-expression *ERF1-V* were again screened by PCR analysis of both the *Bar* gene and the *Hv-ERF* gene. The positive transgenic plants were used to evaluate resistance to powdery mildew. The T_2_ generation individuals derived from the transgenic T_1_ plants were molecular characterized again and re-evaluated of their responses to the powdery mildew to reveal the contribution of the *ERF1-V* to the powdery mildew resistance. At the seedling stage, the powdery mildew resistance levels were evaluated using the detached leaves inoculated with a mixture *Bgt* collected from Eastern China. At the adult stage, the resistance levels were also recorded for the corresponding adult plants in the greenhouse. The resistance levels of the transgenic plants were continuously observed each year in the following T_3_ to T_6_ generation. The level of resistance at the seedling stage was classified as grades 0 to 4 according to the standard of Sheng (1988), and the level of resistance in the adult stage was classified as grades 0 to 9.

### Evaluation of Drought and Salt Tolerance

It was induced from the molecular analysis and resistance evaluation in T_1_ and T_2_ generation that the transgenic plants showed higher level of the powdery mildew resistance. Then, T_3_ and T_4_ generation individuals derived from the transgenic T_2_ plants with improved resistance were evaluated of the powdery mildew resistance, and the homozygous lines without resistance separation in T_5_ generation were selected for drought and salt tolerance evaluation. Healthy and plump seeds of *ERF1-V* transgenic lines and the recipient control Yangmai158 were immersed in water for 10 h at 25°C. The seeds were then transferred to Petri dishes layered with two damp sheets of filter papers. Three days later, the uniform seedlings were further transferred to a 100-well box for culturing with 1/2 Hoagland nutrition solution. This was replaced with fresh solution every 2 days. The seedlings were cultured using 16 h light (25°C)/8 h dark (22°C) cycle with 80% humidity. For drought tolerance evaluation, the seedlings were treated with 20% PEG-6000 containing 1/2 Hoagland solution for 72 h at the three-leaf stage, and then were re-watered with 1/2 Hoagland solution. Ten seedlings were sampled for fresh weight and dry weight before and after re-watering, and the mean values were calculated based on three repeats. For salt tolerance evaluation, the seedlings were treated with 150 mmol/L NaCl-containing 1/2 Hoagland solution at the three-leaf stage. This was replaced with fresh solution every 2 days. At the five-leaf stage, ten seedlings were sampled for fresh and dry weight of the root and the leaf, and the mean values were calculated based on three repeats. All data were statistically analyzed using SPSS 16.0 software (Norusis, 2008).

### Biochemical and Molecular Changes during Drought and Salt Tolerance

The physiological changes and the gene expression analysis were compared between the transgenic plants and Yangmai158. Chlorophyll and malondialdehyde (MDA) content, and superoxide dismutase (SOD) and peroxidase (POD) activity were measured when phenotypic differences were observed between the transgenic plants and Yangmai158 after salt treatment (Rong et al., 2014; Zhu et al., 2014). Gene expression of *TaGSK* (*TaGSK*-F and *TaGSK*-R, Supplementary Table S1), *TaP5CR* (*TaP5CR*-F and *TaP5CR*-R, Supplementary Table S1), *TaHKT* (*TaHKT*-F and *TaHKT*-R, Supplementary Table S1), *TaNHX* (*TaNHX1*-F and *TaNHX1*-R, Supplementary Table S1) and *TaOAT* (*TaOAT*-F and *TaOAT*-R, Supplementary Table S1) involved in the salt or drought tolerance pathway were measured 72 h after PEG-6000 or NaCl treatment in the transgenic plants and Yangmai58. All data were statistically analyzed using SPSS 16.0 software (Norusis, 2008).

### Agronomic Traits Comparisons

The T_5_ generation plants used for drought and salt tolerance evaluation and the derived T_6_ generation plants without resistance separation were used for agricultural traits comparison. The plants were grown in 1.5 m rows spaced 25 cm apart, with 30 seeds planted per row. Agronomic traits, including plant height, flowering time, tiller number, spikelet number and grain size, were compared between the four transgenic lines and the recipient Yangmai158 at different developmental stages. The experiments were conducted in 2 years using T_5_ and T_6_ generation plants, respectively. In each year, ten plants were sampled and measured for one replicate, the experiments were each repeated three times. All phenotypic data were statistically analyzed using SPSS 16.0 software (Norusis, 2008).

## Results

### The response of *ERF1-V* to Biotic/Abiotic Stress and Hormone Treatments

**FIGURE 1:**
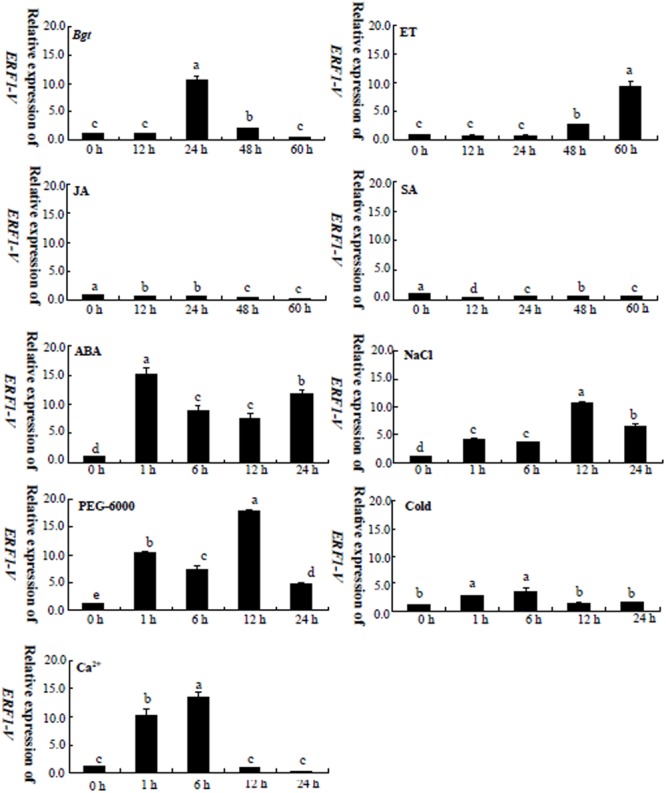
Expression analysis of *ERF1-V* in *Haynaldia villosa* -seedlings treated with biotic stress, abiotic stress and exogenous hormones. *Bgt, Blumeria graminis* f sp. *tirici.* ET: 200 μmol/L Ethephon. JA, 25 μmol/L Jasmonic acid; SA, 5 mmol/L Salicylic acid. ABA: 50 μmol/L. PEG-6000: 16% (W/V) PEG-6000. NaCl: 200 mmol/L NaCL Cold: 4°C, Ca^2+^: 200 mmol/L CaCl2. Different letters indicate significantly different means using the one-way ANOVA LSD analysis (*P* < 0.05).

*Haynaldia villosa*, which contains the *Pm21* gene, confers broad spectrum resistance to powdery mildew (Chen et al., 1995). To identify the resistance related genes and to study the resistance mechanism, the transcription profiles of *H. villosa* were obtained by GeneChip microarray, and the data were compared between the *Bgt* inoculated sample and the uninoculated sample. Many differentially expressed genes were isolated with a signal ratio of more than 2. The ethylene-responsive element-binding factor gene *ERF1-V*, corresponding to the probe of Contig3867, was characterized as a *Bgt* induced gene. The following qRT-PCR was conducted and the results showed that *ERF1-V* was a real *Bgt* responsive gene (**Figure 1**).

AP2/ERF is widely involved not only in the biotic stress response but also in the abiotic stress response, and *H. villosa* shows excellent tolerance to abiotic stress, such as salt and drought (Blanco et al., 1983). So, it is interesting to see whether the *ERF1-V* takes part in the responses of *H. villosa* to abiotic stresses. The qRT-PCR analysis indicated that *ERF1-V* was induced by NaCl, PEG-6000 and cold treatment (**Figure 1**).

The SA, JA, and ethylene (ET) are signal molecules commonly involved in biotic stress response (Yang et al., 2015), and the ABA is a key signal molecule of abiotic stress response (Yoshida et al., 2014). Gene expression analysis showed that *ERF1-V* was up-regulated by ET and ABA, but down-regulated by SA and JA (**Figure 1**). Furthermore, calcium, another signaling molecule, induced the expression of *ERF1-V* (**Figure 1**). These results indicated that the *ERF1-V* might be involved in different signal pathways.

### The Gene Structure and Phylogenetic Analysis of ERF1-V

Among the subfamilies of AP2/ERF, the RAV subfamily has both B3 domain and AP2/ERF domain. The AP2 subfamily possess two repeated domains, while the members of the DREB and ERF subfamilies have a single AP2/ERF domain (Yamasaki et al., 2013). To characterize which subfamily *ERF1-V* belongs to, the full length of *ERF1-V* was cloned by screening the cDNA libraries of *H. villosa*, and the gene structure and phylogenetic analysis of *ERF1-V* was conducted. *ERF1-V* is a 1558 bp gene which contains an 1185 bp ORF corresponding to a 394 amino acid protein (Accession Number: ACN58181). Using the ExPASy software, it was found that ERF1-V contains only a single AP2/ERF conserved domain from 129R to 189P (Supplementary Figure S1a). Analysis by SWISS-MODEL showed that there were three β-sheets and one α-helix in the conserved domain (Supplementary Figures S1a,b). Forty AP2/ERF genes from different subfamilies of 10 species were used for construction of the phylogenetic tree, and the results showed that the *ERF1-V* belonged to the B2 ERF subfamily (Supplementary Figure S2). Multiple sequence alignment using different B2 type genes indicated that the AP2/ERF domain was conserved among the B2 family. Furthermore, conserved ‘A’ and ‘D’ specific to the ERF subfamily were presented in the ERF1-V. However, these two amino acids were ‘V’ and ‘E,’ respectively, in most of the DREB subfamily members (Supplementary Figure S1a). The phylogenetic tree showed that *TaERF1* and *TaERF2* are B2 type, *TaERF3* are B3 type, and *TaERF4* are B1 type (Supplementary Figure S2).

### The Transcriptional Activation Activity Test

**FIGURE 2:**
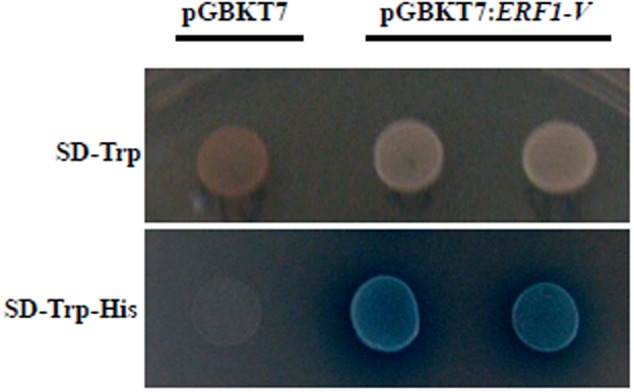
Transcription activation activity test. The yeast cells transformed with the recombinant vector pGBKT7:*ERFI-V* grew well in the *Trp* lacking media, and could be stained blue by *X*-gal in the *Trp* and *His* lacking media.

The transcription factor GAL4, which containing the DNA BD and the transcription activation domain (AD), can bind to the promoter of the downstream genes by BD domain and then activate the downstream gene by AD domain. In the yeast two-hybrid system, if the AD domain of GAL4 was replaced by another protein with transcriptional activation activity, then the transcription of the downstream reporter genes can also be activated. This system is often used to test whether a target gene has transcriptional activation activity. In this study, the *ERF1-V* was inserted into the plasmid *pGBKT7* to replace the AD domain of the GAL4. The recombinant plasmid *pGBKT7:ERF1-V* was then transformed to the yeast cell AH107, and the positive transformed cell grew in the *Trp* and *His* lacking culture media, indicating the reporter gene *His* synthesis gene was activated. The positive cells were stained with X-gal, and the result showed that the reporter gene *LacZ* was activated (**Figure 2**). This indicated that *ERF1-V* had transcriptional activation activity.

### Resistance of the *ERF1-V* Transgenic Plants to Powdery Mildew

**FIGURE 3:**
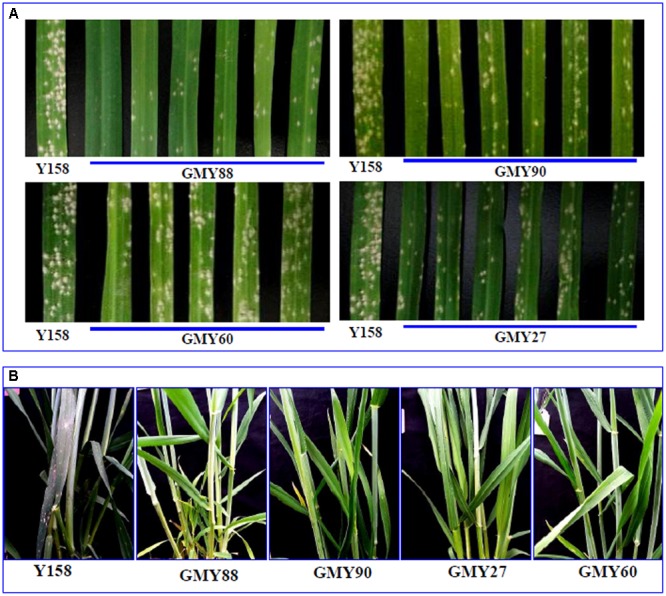
Evaluation of the resistance of the transgenic plants to the powdery mildew. **(A)** The detached leaves of the transgenic plants and the control Yangmail58 were inoculated with the conidiospore of *Bgt* at the seedling stage. Seven days later, the transgenic plants showed increased resistance compared with the control Yangmail58. **(B)** Resistance to *Bgt was* compared at the adult stage in the green house, and the transgenic plants showed higher resistance than the control Yangmail58.

Since *ERF1-V* was induced by *Bgt* in *H. villosa*, the role of the *ERF1-V* in powdery mildew resistance was then evaluated using transgenic plants overexpression *ERF1-V*. Six T_0_ positive transgenic plants were identified as containing both the selective gene *Bar* and the target gene *ERF1-V*. Increased expression of *ERF1-V* was identified in four lines, including GMY27, GMY60, GMY88, and GMY90 (Supplementary Figure S3). The positive T_1_ individuals and the following T_2_ to T_6_ generation of the four T_0_ transgenic lines were used for powdery mildew resistance evaluation both at the seedling stage and the adult stage. At the seedling stage evaluation, the detached leaves of the recipient control Yangmai158 showed high susceptibility with grade 4 infection type after inoculation with *Bgt* on which were covered with high density of *Bgt* spore colony. The detached leaves of T_1_ generation positive individuals showed increased resistance to *Bgt*, on which were covered with reduced density of spore colony. The leaves of transgenic lines GMY88 and GMY90 showed high resistance to *Bgt* with grade 1-2 infection type (**Figure 3A**). At the adult stage evaluation, resistance was evaluated from generations T_2_ to T_6_, and the resistance level of the four transgenic plants ranged from grade 1–5, while the resistance level of the recipient control Yangmai158 ranged from grades 7–8 (**Figure 3B**). Over-expression of the *ERF1-V* gene could enhance the resistance of transgenic plants and their progenies to *Bgt*.

### Tolerance of the *ERF1-V* Transgenic Plants to Drought Stress

**FIGURE 4:**
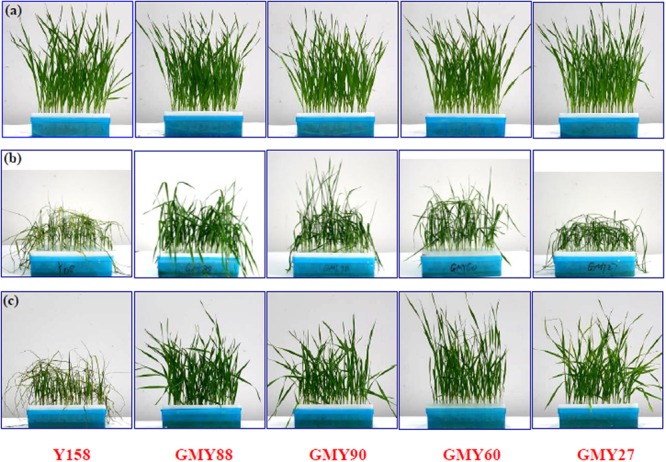
The *ERF1* -*V* overexpression plants showed higher tolerance to drought stress. **(a)** The transgenic plants and Yangmail58 grew well in the 1/2 Hoagland solution, and showed no phenotypic differences. **(b)** The transgenic plants showed higher tolerance to drought than Yangmail58 3 days after 20% PEG-6000 treatment. **(c)** The transgenic plants recovered quickly 1 day after re-watering while the Yangmail58 remained wilted.

*ERF1-V* was rapidly up-regulated after the PEG-6000 treatment, therefore T_5_ generation of the four transgenic lines were tested for their tolerance to drought stress. At the three-leaf stage, the treated plants of the four transgenic lines and Yangmai158 were transferred to 1/2 Hoagland culture solution supplied with 20% PEG-6000. The control plants from the same lines were transferred to the fresh 1/2 Hoagland culture solution. After 3 days, there was no difference between the non-treated Yangmai158 and transgenic plants, and all the plants developed well (**Figure 4a**). While, large differences were observed between the PEG-6000 treated plants Yangmai158 and four transgenic lines. The control Yangmai158 was highly wilted and dry. However, the GMY88 transgenic plants developed normally and the GMY90, GMY60, and GMY27 transgenic lines were only slightly wilted and remained green (**Figure 4b** and Supplementary Figure S4a). All the PEG treated plants were then transferred to fresh 1/2 Hoagland culture solution for re-watering to test the ability of recovery After 1 day, even greater differences were observed between the Yangmai158 and the transgenic plants. The Yangmai158 did not recover and remained dry, while the four transgenic lines completely recovered (**Figure 4c** and Supplementary Figure S4b).

**FIGURE 5:**
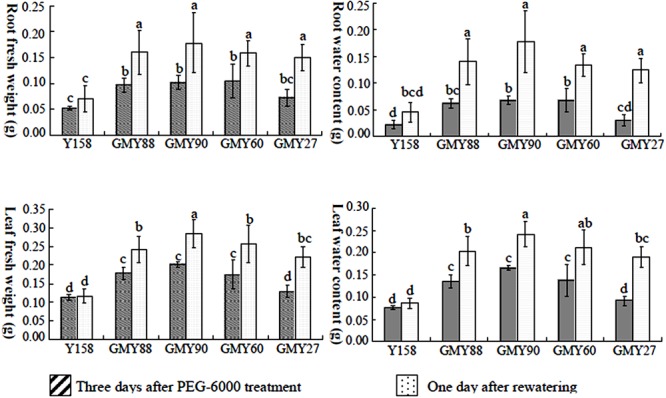
Quantitative analysis of the fresh and dry weight of leaf and root 3 days after PEG-6000 treatment and 1 day after re-watering. Both the fresh weight and the water content of root and leaf were higher in the transgenic lines GMY90. GMY88 and GMY60 than that in the Yangniail58 before and after re-watering. Data are presented as means ± SD and bars marked with different letters indicate significantly different means using the one-way AXOVA LSD analysis (*P* < 0.05).

Biochemical data were collected to study the role of *ERF1-V* in drought tolerance. Three days after PEG-6000 treatment, the leaf and reeo fresh weight of Yangmai158 and the transgenic plants were significantly difference (**Figure 5**). One day after re-watering, the four transgenic plants recovered well and there was a dramatic increase in fresh weight, while the fresh weight of Yangmai158 did not significantly change (**Figure 5**). Drought tolerance and recovery of the transgenic plants were improved by *ERF1-V*.

### Tolerance of the *ERF1-V* Transgenic Plants to Salt Stress

**FIGURE 6:**
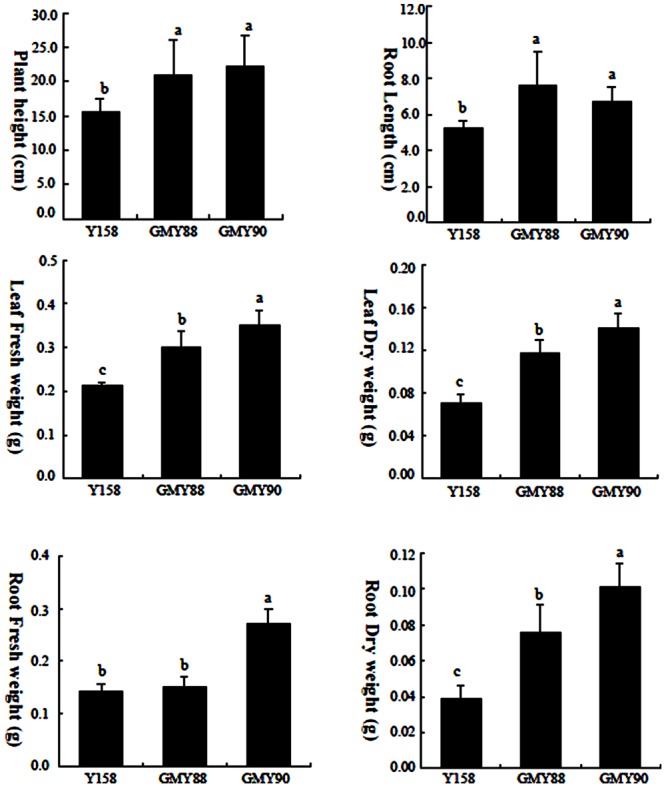
Quantitative analysis of the plant height, root length, fresh and dry weight of leaf and root of the transgenic plants and Yangmail58 after 150 mmol/L NaCl treatment. The transgenic plant; showed increased plant height, root length, leaf fresh weight, leaf dry weight and root dry weight than Yangmail58, respectively. Data are presented as means ± SD and bars marked with different letters indicate significantly different means using the one-way ANOVA LSD analysis (*P* < 0.05).

*ERF1-V* was rapidly up-regulated after the NaCl treatment. Therefore, the transgenic lines GMY88 and GMY90, which showed the best drought stress tolerance among the four lines, were further used to evaluate tolerance to salt stress. There was no significant difference between the Yangmai158 and the transgenic lines before NaCl treatment. However, there were significant differences between the Yangmai158 and the transgenic plants after NaCl treatment for plant height, root length, fresh weight and dry weight of leaf, and dry weight of root (**Figure 6** and Supplementary Figure S5). These results indicated that *ERF1-V* could enhance the tolerance of wheat to salinity.

### The Mechanism of Drought and Salt Resistance Mediated by *ERF1-V*

Transcription factors can regulate many downstream genes participating in osmosis adjustment and detoxification. To elucidate pathways activated by *ERF1-V*, the biochemical data and gene expression pattern of transgenic line GMY90, which showed the best tolerance to salinity and drought, were collected and compared with that of Yangmai158.

**FIGURE 7:**
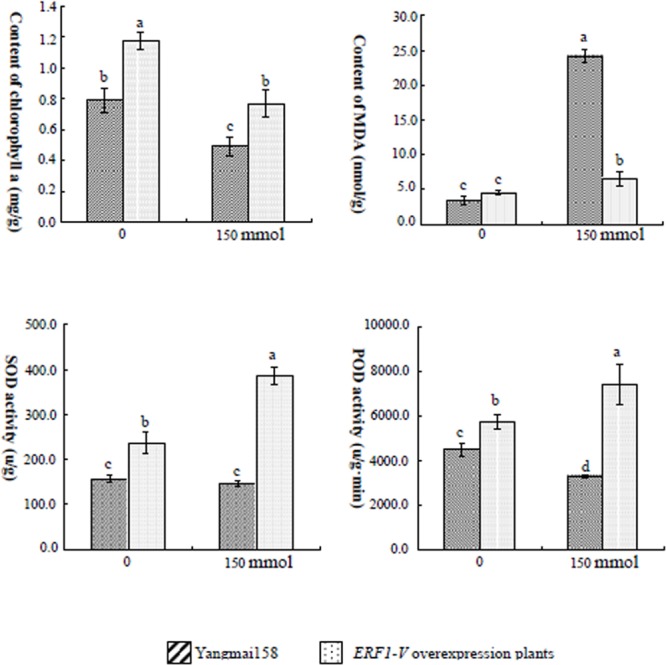
Chlorophyll a+b and MDA content, and SOD and POD activity of transgenic plants and Yangmai158 7 days after NaCl treatment. The transgenic plants showed higher content of chlorophyll a+b content, higher SOD and POD activity, but lower MDA accumulation than Yangmail58. Data are presented as means ± SD and bars marked with different letters indicate significantly different means using the one-way ANOVA LSD analysis (*P* < 0.05).

Abiotic stresses can lead to the generation of reactive oxygen species (ROSs) which induce secondary oxidative stress. Abiotic stress tolerant species are usually efficient at ROSs scavenging (Mittler, 2002). In this study, the activity of the superoxide dismutase (SOD) and peroxidase (POD), two crucial antioxidant enzymes, significantly increased in GMY90 when treated with NaCl compared with the untreated control plants. No significant difference in SOD and POD activity was observed between the treated and untreated Yangmai158 plants (**Figure 7**).

Higher MDA content reflected greater membrane damage. In Yangmai158, MDA content increased dramatically after NaCl treatment, while there was no such increase in the NaCl-treated transgenic plants GMY90 (**Figure 7**). This result indicated that the abiotic stress tolerance was increased by over-expression of *ERF1-V* which then reduced the membrane damaging. Lower chlorophyll content can be an indicator of extensive damage. Chlorophyll content was higher in the GMY90 plants compared with Yangmai158 following salt stress (**Figure 7**).

**FIGURE 8:**
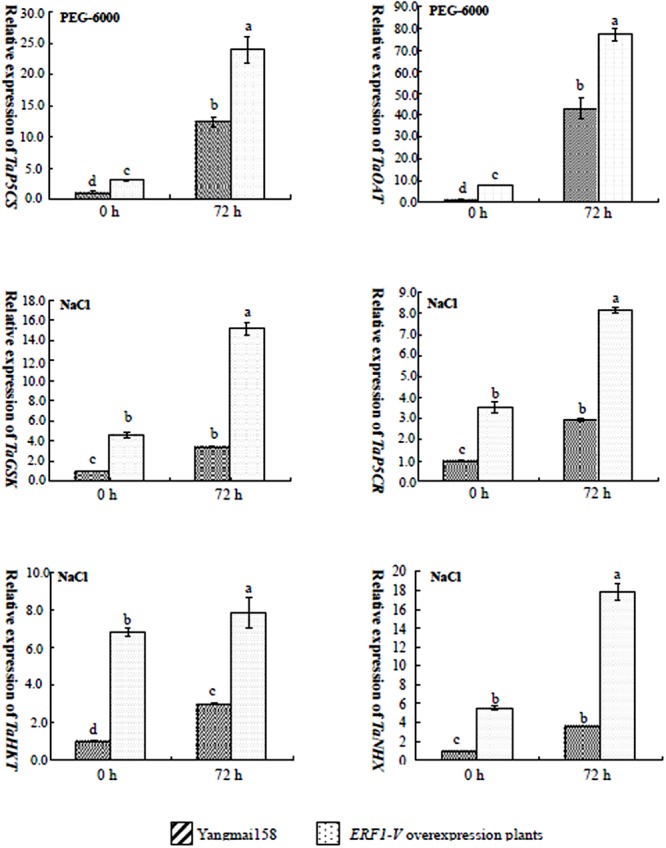
Expression patterns of important marker genes involved in stress response pathways in the transgenic plants and Yangmail58 before and after NaCl or PEG-6000 treatment. The *TaP5CS. TaOAT. TaGSK, TaHKT*, and *TaNHX* genes were induced by NaCl or PEG-6000 both in the Yangniail58 and in the transgenic plants. However, in the transgenic plants the stress responsive genes showed higher expression level under stresses. Data are presented as means ± SD and bars marked with different letters indicate significantly different means using the one-way ANOVA LSD analysis (*P* < 0.05).

The expression of six stress responsive genes, including *P5CR* and *OAT* involved in the Proline (Pro) synthesis related to osmosis adjustment, *GSK* (glycogen synthase kinase 3) involved in salt signaling, *HKT* (Na^+^ transporter gene) and *NHX* (vacuolar membrane transporters) involved in the cation transport, were analyzed by qRT-PCR. The results showed that *GSK, P5CR, HKT*, and *NHX* were induced by NaCl in the Yangmai158 and GMY90, but in GMY90 the expression levels of four genes were higher than that in the Yangmai158 (**Figure 8**). *P5CR* and *OAT* were induced by PEG-6000 in both materials, but in GM90, the expression levels were higher than in the Yangmai158 (**Figure 8**).

### Agronomic Trait Observation of *ERF1-V* Transgenic Plants

**FIGURE 9:**
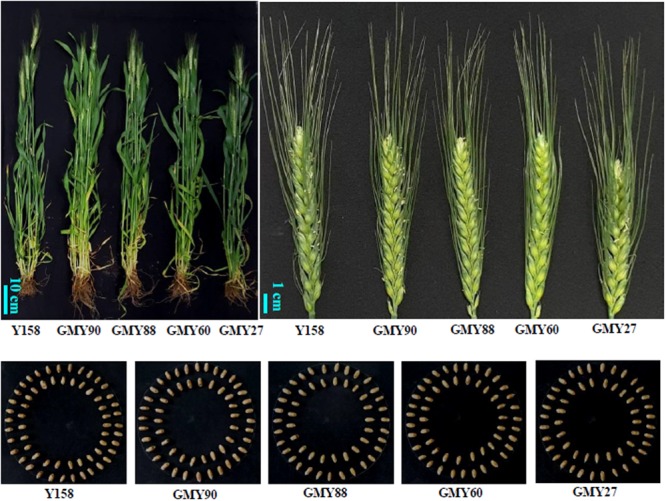
The phenotypes of adult plants, spikes and the seeds of the transgenic plants and Yangmail58.

The over-expression of TFs usually has negative effects on plant development. In this study, to find out whether the over-expression of *ERF1-V* could affect the growth of wheat, several main agronomic traits, including plant height, flowering time, tiller number, spikelet number and grain size, were compared between the transgenic plants and the wild recipient Yangmai158. The transformation of the *ERF1-V* did not impact flowering time, spikelet number, tiller number and grain size (**Figure 9** and Supplementary Figure S6). Yet, plant height of GMY88 and GMY90 were slightly increased, and spike length of the GMY27 was slightly reduced.

## Discussion

This study isolated and characterized of an AP2/ERF transcription factor *ERF1-V* from wild *H*. *villosa* which could rapidly respond to multiple biotic and abiotic stresses. Functional analysis indicated that *ERF1-V* positively regulate wheat powdery mildew resistance, and drought and salt stress tolerance. AP2/ERF transcription factors were the most widely identified genes which had important roles in regulating plant responses to abiotic stresses. Some AP2/ERF genes were found to enhance single stress tolerance, for example, *GmERF7, LcDERB2, LcERF054*, and *StDERB1* regulate salinity tolerance, while *JERF1, JERF3, OsERF4a*, and *AtDREB1A* regulate drought tolerance. Some AP2/ERF genes were found to enhance tolerance to several stresses, for example, *TERF3* and *OsDREB2A* in rice, *SodERF3, SsDREB*, and *GmERF4* in tobacco, *LcDERB3a* and *VrDERB2A* in *Arabidopsis*, and *EaDREB2* in sugarcane could enhance tolerance to both drought and salinity stress (Xu et al., 2011). Some AP2/ERF genes were found to be involved in the abiotic and biotic stresses responses simultaneously, such as *HvRAF* improved pathogen resistance and salinity tolerance in *Arabidopsis, CaPF1* enhanced multiple pathogens resistance and heat tolerance, *GmERF3* increased drought and salinity tolerance and disease resistance, and *TSRF1* enhanced osmotic and drought tolerance and pathogen resistance (Jung et al., 2007; Zhou et al., 2008; Zhang et al., 2009). Up to date, only a few AP2/ERF genes have been analyzed in wheat. *TaERF1* improved tolerance to salt, drought and cold stress, and increased resistance to *P. syringae* in *Arabidopsis* (Xu et al., 2007), *TaERF3* enhanced tolerance to drought and salt (Rong et al., 2014), *TaPIE1* (pathogen-induced ERF1) enhanced resistance to necrotrophic pathogen *Rhizoctonia cerealis* and freezing stress in wheat (Zhu et al., 2014), while *TaERF4* was reported to be a repressor of salinity tolerance (Dong et al., 2012). So, exploration and elucidation of more AP2/ERF genes from wheat and its wild species can enrich AP2/ERF resources. *ERF1-V* provides a strong potential target gene for promoting the development of wheat genetic engineering breeding.

The environmental stress responsive genes were divided into two groups, one group was the ‘functional proteins’ and the other is the ‘regulatory proteins’ (Shinozaki et al., 2003; Wang et al., 2016). The ‘functional proteins’ include the proteins as detoxification enzymes and osmotic adjustment proteins, which function to cells protection from damage, however, significant enhancement of stress tolerance cannot be easily achieved by modifying a single gene. The ‘regulatory proteins’ includes TFs and protein kinases, which function to regulate signal transduction and modify expression of multiple downstream genes, and sometime one gene is enough to protect the plants against the severe stress. TFs, a kind of ‘regulatory protein’, can bind to specific *cis*-elements in the promoter of downstream genes and change their expression level. ERF can bind to two similar *cis*-elements, such as GCC box in the promoter of the pathogenesis-related gene and DRE motif in the promoter of the dehydration genes (Zhu et al., 2014). More interesting was that some ERF, such as *ERF1* in *Arabidopsis*, could bind to different type of *cis*-elements under different stresses (Cheng et al., 2013). So over-expression *ERF* genes has advantage to improve both biotic stress and abiotic stress. In this study, *ERF1-V* perhaps binds to various *cis*-elements of a set of downstream genes involved in powdery mildew resistance, salt and drought stress responses, so modifying single *ERF1-V* gene could improve both resistance to disease and tolerance to drought and salt tolerance.

Due to the ability to affect multi gene expression, in some cases, the transgenic plants over-expression TFs showed multi traits changes if their regulated genes were involved in different physiological processes. Dwarf phenotype was observed in transgenic rice over-expressing *TaDREB1* (Shen et al., 2003), and slower growth, delayed flowering and lower grain yields were found to be related with over-expression *TaDREB2* and *TaDREB3* in wheat (Morran et al., 2011). In this study, over-expression of *ERF1-V* did not impact flowering time, spikelet number, tiller number and grain size. Although plant height of GMY90 and GMY88 was slightly increased and spike length of GMY27 was slightly reduced, such kind of change was not occurred in all the tested transgenic lines. The most important was that none of transgenic plants showed serious abnormal developmental changes. So the *ERF1-V* perhaps participated in regulating the genes which was mostly involved in the abiotic and biotic stress response, while not regulating those genes involved in the growth and development process. The wheat transgenic lines with normal phenotype and increased drought and salt tolerance were successfully selected in the high transgenic generation, which indicates that *ERF1-V* gene has huge potential for wheat breeding.

The expression of *ERF1-V* could be induced by exogenous ET, ABA and Ca^+^, indicates that it might function through different signal transduction pathways involved in biotic and abiotic tolerance. As we knew, SA, JA, ET, ABA, and Ca^+^ are the signal factors that participating widely in the biological pathways with cooperative or antagonistic functions as reported. Some abiotic stress tolerance related *ERFs* were found to play roles through ET pathway, such as *TERF2/LeERF2* in tomato (Zhang and Huang, 2010), and *TaPIE1* in wheat (Zhu et al., 2014). In our previous study, it was found that powdery mildew resistance of *H. villosa* was tightly related to up-regulating of ethylene biosynthesis genes and signal transduction genes including *ERF1-V* (Cao et al., 2006). ABA is a key plant hormone involved in diverse physiological and developmental processes, including abiotic stress responses and the regulation of stomatal aperture and seed germination (Burla et al., 2013). Calcium is a second messenger important to biotic and abiotic stresses responsive pathways, and the amount of calcium in the cytosol was found to be increased under salt stress and pathogen inoculation (Kader and Lindberg, 2010). So, ET, ABA and calcium signal pathways may regulate *ERF1-V* cooperatively which leading to improvement of powdery mildew resistance and abiotic stress tolerance.

Osmotic balance is important for plant survival under the serious drought and salinity stress, and proline (Pro) accumulation was found be usually occur in the abiotic tolerant species (Cheng et al., 2013). Based on the comparison between Yangmai158 and GMY90 showing best tolerance to salinity stress, it was proposed that over-expression of *ERF1-V* might contribute to the osmosis adjustment for the *P5CS* and *OAT*, two crucial genes involved in the Pro synthesis through Glu or ornithine pathways (Delauney and Verma, 1993), was up-regulated in GMY90 under stresses. Detoxification is another important pathway often used by the abiotic stress tolerant species, and ROS scavenge mediated by SOD and POD is extremely important for cell protection (Mittler, 2002). In this study, over-expression *ERF1-V* could enhance SOD and POD activity simultaneously which contributing to the stress tolerance. It was also found that expression of salt tolerance response genes including *GSK, HKT*, and *NHX* was highly induced in the transgenic plants after salt treatment. So, it was proposed that *ERF1-V* functions as a ‘regulatory gene’ when wheat was under the severe stresses, which can change the expression level of several ‘functional genes’ and alter the content of several metabolism compounds.

## Conclusion

Although our results implied that *ERF1-V* induced biochemical and molecular changes during drought and salt tolerance in transgenic plants, the roles of ET, ABA and Ca^2+^ signaling pathways in wheat powdery mildew resistant and drought and salt stress tolerance remains unclear due to the regulatory mechanisms underlying the cross-talk between these signaling pathways are complicated. Therefore, the molecular mechanism through which *ERF1-V* functions to improve resistance to disease and tolerance to abiotic stresses requires further study.

## Accession Number

The accession number for *ERF1-V* is ACN58181.

## Author Contributions

LX and AC designed the experiment, performed the data analysis and wrote the manuscript.XWdesigned the experiment to screen the pathogen induced genes in *Haynaldia villosa*. XyW cloned the *ERF1-V* gene and detected the responses of *ERF1-V* to biotic and abiotic stresses. JX conducted the bioinformatics analysis. ML developed the transgenic plants and XjW evaluated the powdery mildew resistance using the T_1_ generation of the positive transgenic plants. JL analyzed the expression level of the abiotic stress related gene in the transgenic plants. CC and ZD evaluated the tolerance of the transgenic plants to drought and salinity stresses. RZ and WY collected the data of the agronomic traits and evaluated the powdery mildew resistance in the field using high generations of the transgenic plants.

## Conflict of Interest Statement

The authors declare that the research was conducted in the absence of any commercial or financial relationships that could be construed as a potential conflict of interest. The reviewer NK and handling Editor declared their shared affiliation.

## Footnotes

1 https://prosite.expasy.org

2 http://swissmodel.expasy.org

3 www.megasoftware.net
