# Supplementary material for: Overexpression of ERF1-V from Haynaldia villosa Can Enhance the Resistance of Wheat to Powdery Mildew and Increase the Tolerance to Salt and Drought Stresses
Source: Front Plant Sci. 2017 Nov 29;8:1948. doi: 10.3389/fpls.2017.01948 (PMC5712803; doi:10.3389/fpls.2017.01948)
Supplement: Supplementary file 1 [file Image_1.pdf]

Supporting Information

Supporting Table S1. Designed primers

Supporting Figure S1. Amiono acid sequence alignment of the conserved AP2 domain in the B2 type ERF proteins and the three dimensional structure of the AP domain in the ERF1-V.

(a) Multiple alignment of the conserved AP2 domain in the B2 type ERF proteins. The RAP2.3 (NM_112550) and RAP2.12 (NP_175794) of *Arabidopsis thaliana,* JERF1 (AY044235) and JERF3 (EU910896) from *Solanum lycopersicum*, GmERF3 (EU681278) of *Glycine max* and GhERF2 (AY781117) of *Gossypium hirsutum* were downloaded from the NCBI and used to make the multiple alignment with ERF1-V. The red asterisk above the sequences indicate the conserved amino acid 'A' and 'D' specific to *ERF* subfamily genes which was distinct from that of the *DREB* subfamily genes. Three β-sheets and one α-helix were underlined.

(b) Three dimensional structure of the AP domain in the ERF1-V.

Supporting Figure 2. The phylogenetic analysis of ERF1-V and related proteins by MEGA6.

ERF1-V was classified to the B2 type ERF subfamily. The sequence of AtERF4 (AY140030), AtERF7 (AB032201), AtERF3 (AB008105), AtERF1 (NM_113225), AtERF110 (NP_199819), ABR1 (NM_125871), RAP2.3 (NM_112550), RAP2.12 (NP_175794), RAP2.6 (AY114582), CRF1 (NP_192852), CRF5 (NP_182154), SHN2 (NM_121157), SHN3 (NM_122448), At5g67180 (TOE3,NM_126118), At4g37750 (ANT, NM_119937), At2g28550 (RAP2.7, NM_001202696), At1g71450 (FUF1, NM_105814), At1g78080 (RAP2.4, NM_106457), At5g25810 (TNY, NM_122482), At1g50680 (NM_103950), At1g13260 (RAV1, NM_101197), At1g68840 (RAV2, NM_001198423) were from *Arabidopsis thaliana*, SodERF3 (AM493723) was from

Saccharum officinarum, JERF1 (AY044235), JERF3 (EU910896), LeERF2 (NM_001247379), LeERF3 (AY192369) and TSRF1 (AF494201) were from *Solanum lycopersicum*, TaERF1 (AY271984), TaERF2 (AY271985) and TaERF3 (EF570122) were from *Triticum aestivum*, GmERF3 (EU681278) was from *Glycine max*, GhERF2 (AY781117) was from *Gossypium hirsutum*, CaERFLP1 (AY529642) was from *Capsicum annum*, NtERF5 (AY655738) and Tsi1 (AF058827) were from *Nicotiana tabacum*, Sub1A-1 (AAZ06252) was from *Oryza sativa*, and TiERF1 (EF570121) was from *Thinopyrum intermedium*.

Supporting Figure S3. Identification of the positive *ERF1-V* transgenic plants.

(a) Positive transgenic plant identification using the *Bar* gene by PCR analysis.

(b) Gene expression analysis in the identified transgenic plants by RT-PCR analysis.

CK: plasmid DNA; Y158: recipient Yangmai158

Supporting Figure S4. Evaluation of the transgenic plants to drought tolerance.

(a) The seedlings of the *ERF1-V* transgenic plants were less wilted than Yangmai158 three days after PEG-6000 treatment.

(b) The seedlings of the *ERF1-V* transgenic plants recovered quickly one day after re-watering while Yangmai158 was still wilted.

Supporting Figure S5. Evaluation of the transgenic plants to salt tolerance.

The *ERF1-V* over-expression plants showed higher tolerance to salt stress. (a), (b). The seedlings and roots cultured in the Hoagland nutrition. (c), (d). The seedlings and roots cultured in 150mmol/L NaCl.

Supporting Figure S6. Measurement of plant height, effective tiller number, spike length, spike number of the transgenic plants and Yangmai158. Data are presented as means ± SD, and bars marked with different letters indicate significantly different means using the one-way ANOVA LSD analysis ($P < 0.05$).

Supporting Table

Table S1. Designed primers

	Primer Name	Primer Sequence (5' to 3')
PCR primers for cDNA library screening	*ERF1-V*-F	CTCTCCCAAGATGACTTCAG
	ERF1-V-R	CATAAGAAACCTCGTCCAAG
Primers for pGBKT7:*ERF1-V* vector construction	*ERF1-V-EcoR*I-F	CCGGAATTCATGTGCGGCGGCGCGATCCT
	*ERF1-V-Sma*I-R	TCACCCGGGTCAGAAACCACAAACGGGCA
Primers for pAHC:*Hv-ERF* vector construction	*ERF1-V-Sma*I-F	TCCCCCGGGATGTGCGGCGGCGCGATCCT
	*ERF1-V-Sac*I-R	ACCGAGCTCTCAGAAACCACAAACGGGCA
Primers for *ERF1-V* detection in the transgenic plants	*Ubi*-F	CCCTGTTGTTTGGTGTTAC
	ERF1-V-TR	TTAAGTCATCTTGGGAGAGG
Primers for *Bar* detection in the transgenic plants	*Bar*-F	CAGGAACCGCAGGAGTGGA
	Bar-R	AAACCCACGTCATGCCAGTTC
Primers for semi-quantitative RT-PCR of *ERF1-V* in the transgenic plants	*ERF1-V*-RT-F	AAGAACCAATTCAGGGGTAT
	ERF1-V-RT-R	TTGGTTCCTCTGGAAAGTTA
qRT-PCR primers for *ERF1-V* after stresses and hormone treatments	*ERF1-V*-QRT-F	AGCACAGGAACCTACCGTCAT
	ERF1-V-QRT-R	CCCAAGTCAGAACAGCCAAA
qRT-PCR primers for the abiotic stress tolerance related genes	*TaNHX1*-F	GGGAGTGGTTACTGGTCTGC
	TaNHX1-R	ATGACAATGTCGCAAAAGCA
	TaP5CR-F	AATAGAGGCCATGGCTGATG
	TaP5C -R	AGGGGAAGTGACCTGATCCT
	TaHKT-F	CAAAGGTGAAGGAGCTGAGG
	TaHKT-R	GAGCTGAGCCCATCAAAGAC
	TaGSK-F	GGCAAGCAAAACAGACCATT
	TaGSK-R	TCAAGAACTCGCATCGTTTG
	TaOAT-F	GGCTTACACTTAGTTCCAGAGC
	TaOAT-F	TCATAACCCCATTTTCTTGCCA
Primers for the internal control gene	*Tubulin*-F	AGAACACTGTTGTAAGGCTCAAC
	Tubulin-R	GAGCTTTACTGCCTCGAACATGG

Supporting Figure S1

(a)

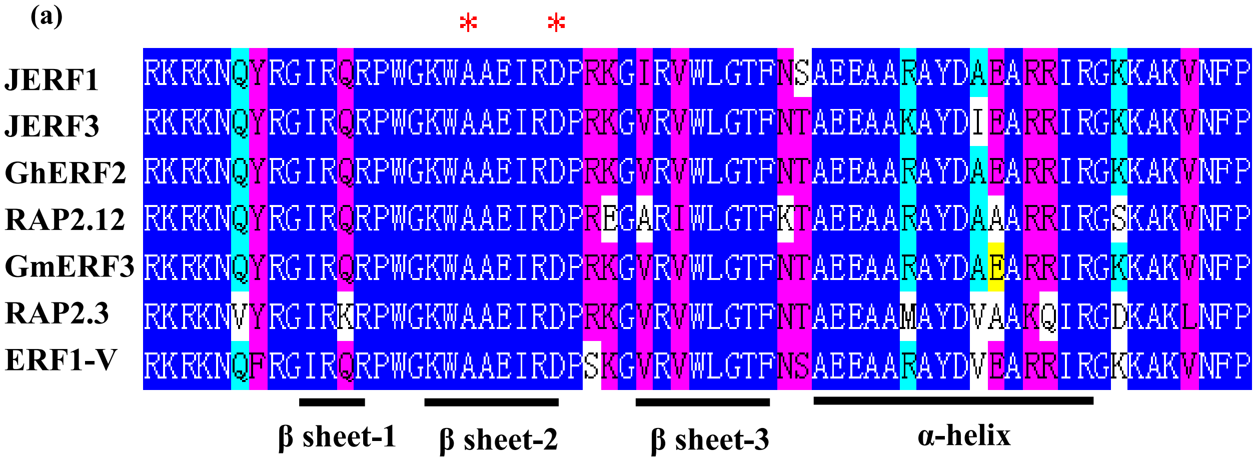

(b)

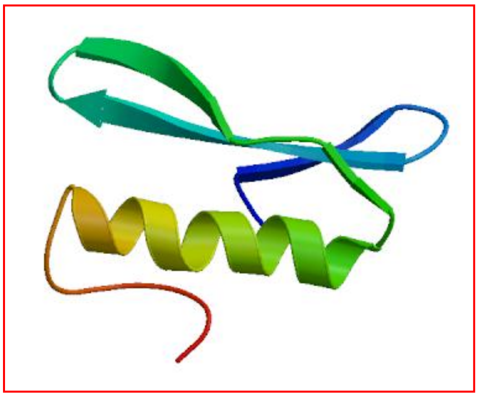

Supporting Figure S1. Amiono acid sequence alignment of the conserved AP2 domain in the B2 type ERF proteins and the three dimensional structure of the AP domain in the ERF1-V.

(a) Multiple alignment of the conserved AP2 domain in the B2 type ERF proteins. The RAP2.3 (NM_112550) and RAP2.12 (NP_175794) of *Arabidopsis thaliana*, JERF1 (AY044235) and JERF3 (EU910896) from *Solanum lycopersicum*, GmERF3 (EU681278) of *Glycine max* and GhERF2 (AY781117) of *Gossypium hirsutum* were downloaded from the NCBI and used to make the multiple alignment with ERF1-V. The red asterisk above the sequences indicate the conserved amino acid ‘A’ and ‘D’ specific to *ERF* subfamily genes which was distinct from that of the *DREB* subfamily genes. Three β-sheets and one α-helix were underlined. (b) Three dimensional structure of the AP domain in the ERF1-V.

Supporting Figure S2

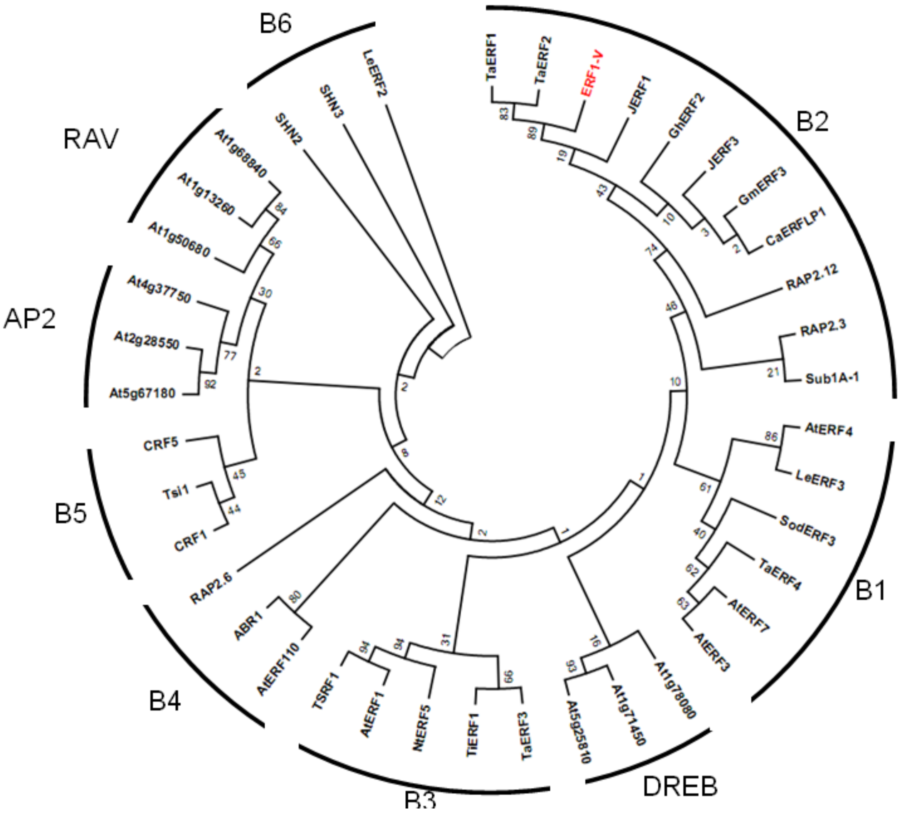

Supporting Figure S2. The phylogenetic analysis of ERF1-V and related proteins by MEGA6. ERF1-V was classified to the B2 type ERF subfamily. The sequence of AtERF4 (AY140030), AtERF7 (AB032201), AtERF3 (AB008105), AtERF1 (NM_113225), AtERF110 (NP_199819), ABR1 (NM_125871), RAP2.3 (NM_112550), RAP2.12 (NP_175794), RAP2.6 (AY114582), CRF1 (NP_192852), CRF5 (NP_182154), SHN2 (NM_121157), SHN3 (NM_122448), At5g67180 (TOE3,NM_126118), At4g37750 (ANT, NM_119937), At2g28550 (RAP2.7, NM_001202696), At1g71450 (FUF1, NM_105814), At1g78080 (RAP2.4, NM_106457), At5g25810 (TNY, NM_122482), At1g50680 (NM_103950), At1g13260 (RAV1, NM_101197), At1g68840 (RAV2, NM_001198423) were from *Arabidopsis thaliana*, SodERF3 (AM493723) was from *Saccharum officinarum*, JERF1 (AY044235), JERF3 (EU910896), LeERF2 (NM_001247379), LeERF3 (AY192369) and TSRF1 (AF494201) were from *Solanum lycopersicum*, TaERF1 (AY271984), TaERF2 (AY271985) and TaERF3 (EF570122) were from *Triticum aestivum*, GmERF3 (EU681278) was from *Glycine max*, GhERF2 (AY781117) was from *Gossypium hirsutum*, CaERFLP1 (AY529642) was from *Capsicum annum*, NtERF5 (AY655738) and Tsi1 (AF058827) were from *Nicotiana tabacum*, Sub1A-1 (AAZ06252) was from *Oryza sativa*, and TiERF1 (EF570121) was from *Thinopyrum intermedium*.

Supporting Figure S3

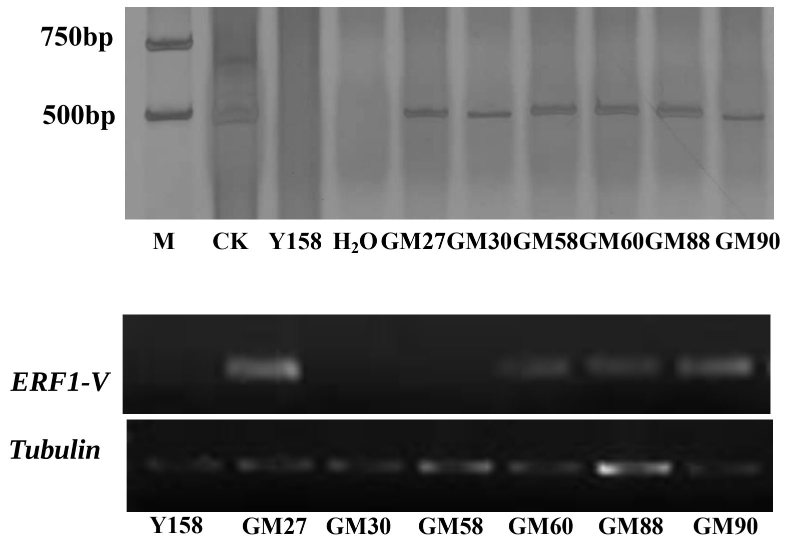

Supporting Figure S3. Identification of the positive *ERF1-V* transgenic plants.

(a) Positive transgenic plant identification using the *Bar* gene by PCR analysis.

(b) Gene expression analysis in the identified transgenic plants by RT-PCR analysis.

CK: plasmid DNA; Y158: recipient Yangmai158

Supporting Figure S4

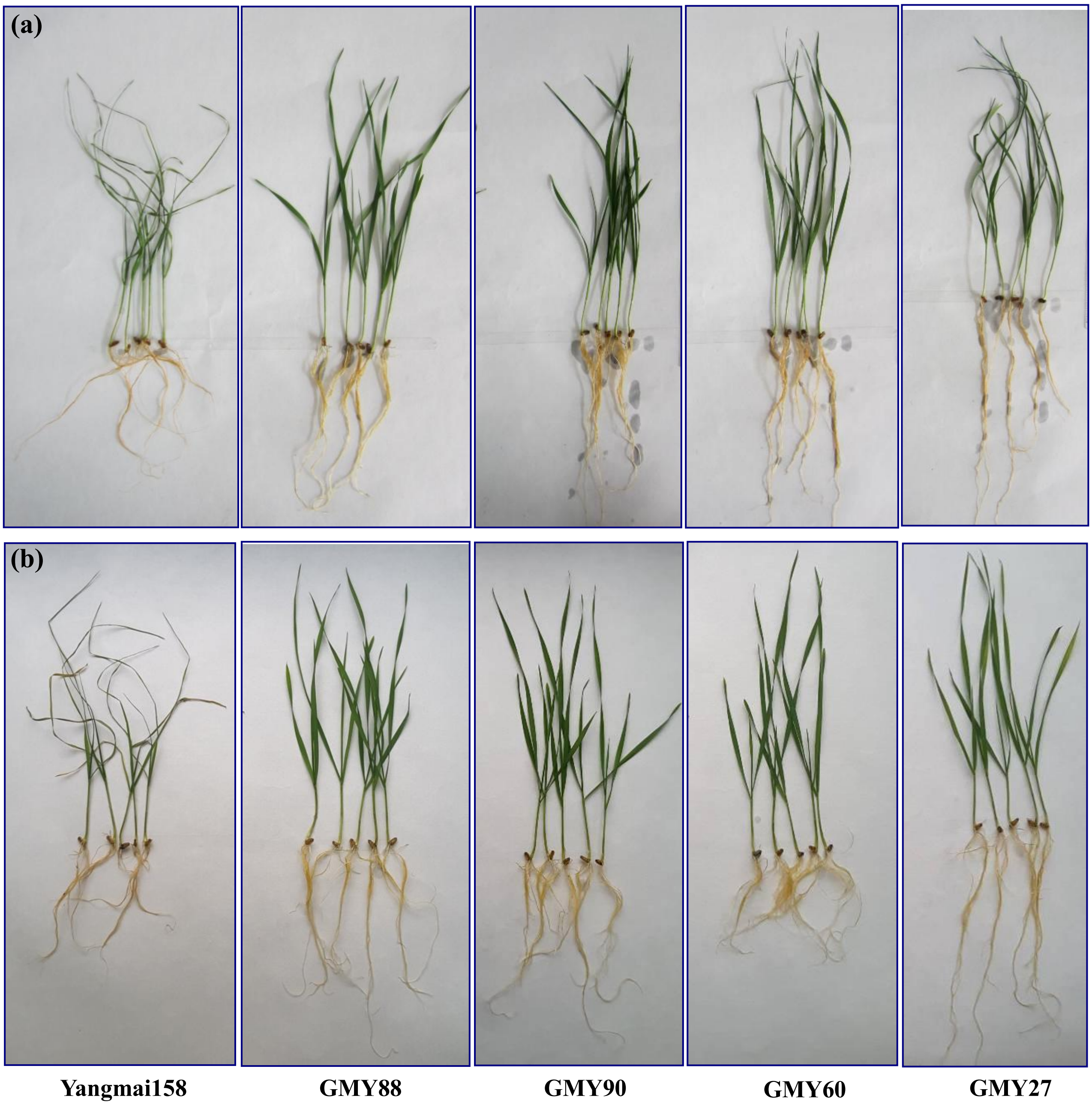

Supporting Figure S4. Evaluation of the transgenic plants to drought tolerance.

(a) The seedlings of the *ERF1-V* transgenic plants were less wilted than Yangmai158 three days after PEG-6000 treatment.

(b) The seedlings of the *ERF1-V* transgenic plants recovered quickly one day after re-watering while Yangmai158 was still wilted.

Supporting Figure S5

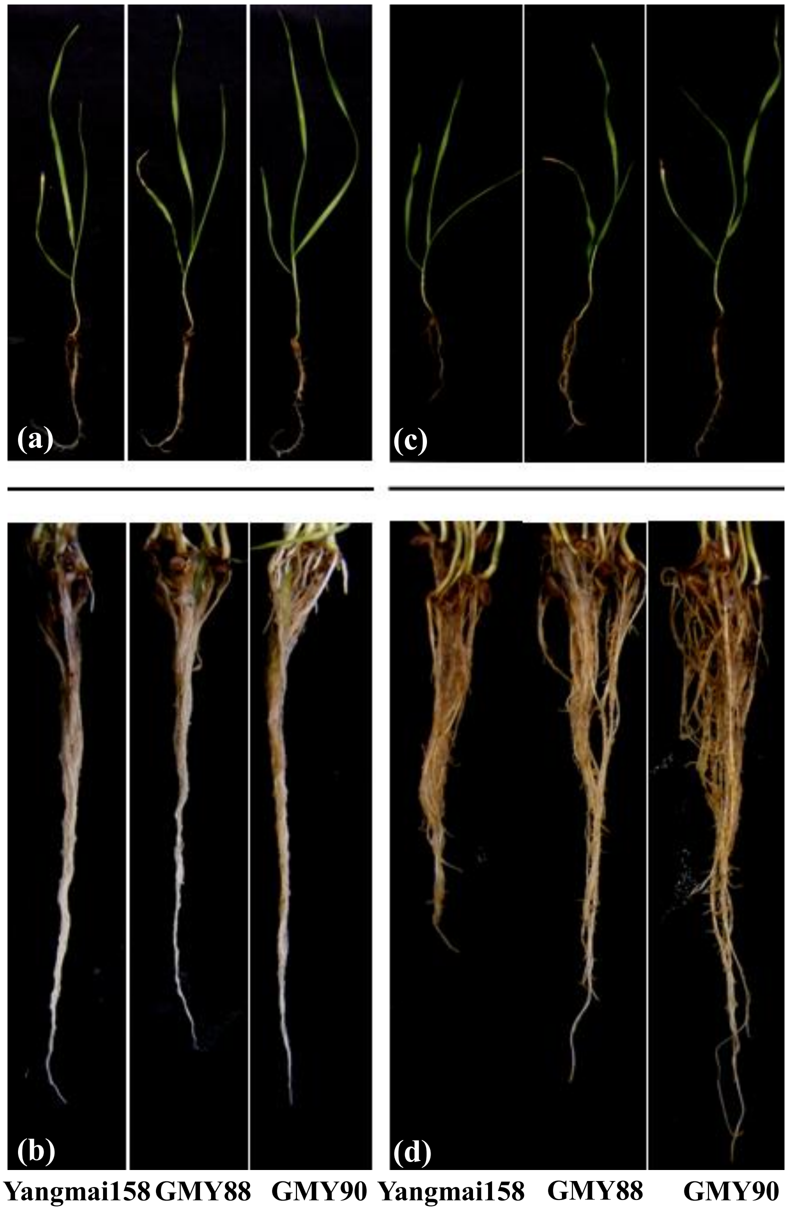

Supporting Figure S5. Evaluation of the transgenic plants to salt tolerance. The *ERF1-V* over-expression plants showed higher tolerance to salt stress.

(a), (b). The seedlings and roots cultured in the Hoagland nutrition.

(c), (d). The seedlings and roots cultured in 150mmol/L NaCl.

Supporting Figure S6

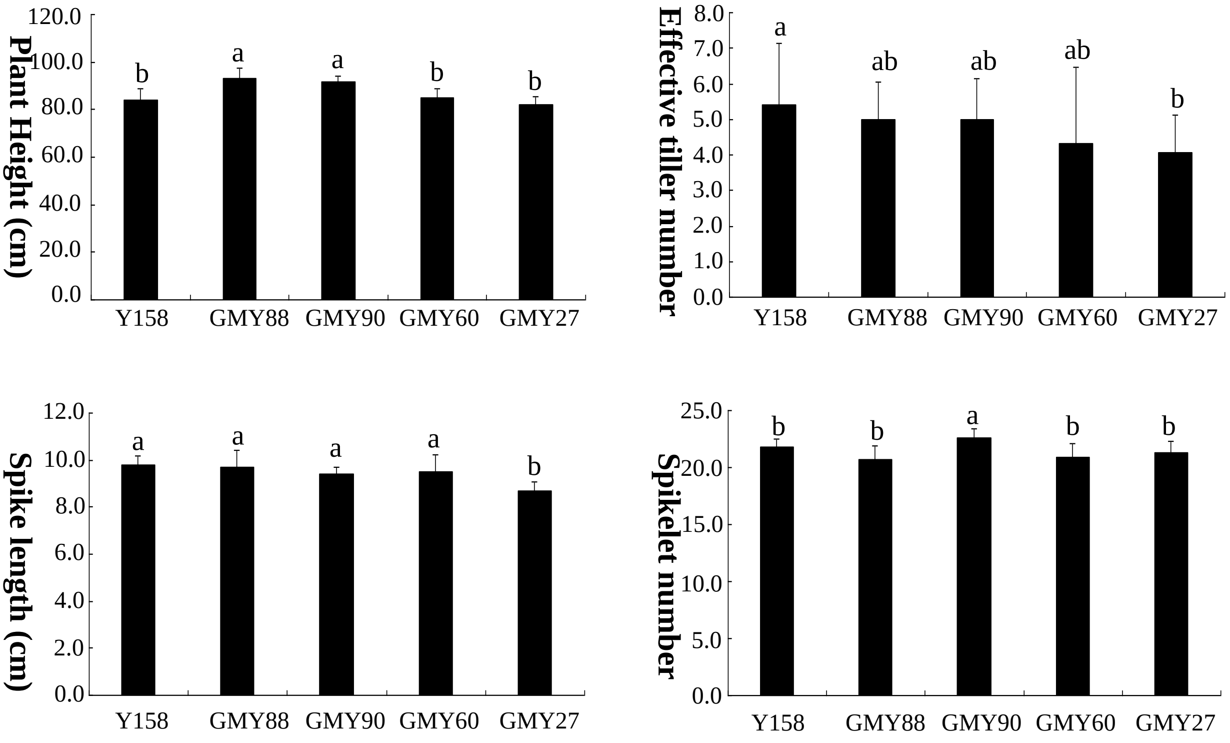

Supporting Figure S6. Measurement of plant height, effective tiller number, spike length, spike number of the transgenic plants and Yangmai158. Data are presented as means±SD, and bars marked with different letters indicate significantly different means using the one-way ANOVA LSD analysis (P <0.05).
